# Fast QRS Detection with an Optimized Knowledge-Based Method: Evaluation on 11 Standard ECG Databases

**DOI:** 10.1371/journal.pone.0073557

**Published:** 2013-09-16

**Authors:** Mohamed Elgendi

**Affiliations:** Department of Computing Science, University of Alberta, Edmonton, Canada; University of Minnesota, United States of America

## Abstract

The current state-of-the-art in automatic QRS detection methods show high robustness and almost negligible error rates. In return, the methods are usually based on machine-learning approaches that require sufficient computational resources. However, simple-fast methods can also achieve high detection rates. There is a need to develop numerically efficient algorithms to accommodate the new trend towards battery-driven ECG devices and to analyze long-term recorded signals in a time-efficient manner. A typical QRS detection method has been reduced to a basic approach consisting of two moving averages that are calibrated by a knowledge base using only two parameters. In contrast to high-accuracy methods, the proposed method can be easily implemented in a digital filter design.

## Introduction

According to the World Health Organization, cardiovascular diseases are the number one cause of death worldwide [1]. An estimated 17.3 million people died from cardiovascular diseases in 2008, representing 30% of all global deaths [1]. Thus, recently, medical researchers have placed significant importance on cardiac health research. This has produced a strong focus on preventative, medicinal, and technological advances. One such research pathway is leading researchers toward improving the conventional cardiovascular-diagnosis technologies used in hospitals, clinics and the home.

The most common clinical cardiac test is electrocardiogram (ECG) analysis. It represents a useful screening tool for various cardiac abnormalities because it is simple, risk-free, and inexpensive [2]. Therefore, the analysis of ECG signals has been extensively investigated over the past two decades. Many attempts have been made to find a satisfying universal solution for QRS complex detection, including the Pan and Tompkins algorithm [3], which has been used extensively in the literature for beat detection. The current advances in battery-driven devices such as smartphones and tablet computers have made these technologies invariably part of daily life, even in developing countries [4]. The advances have also increased the possibility of implementing more sophisticated algorithms such as the Pan and Tompkins method [3] in smartphones. However, there is a significant trade-off as there will always be a power-consumption limitation in processing ECG signals on battery-operated devices.

Analyzing real-time ECG signals collected by a battery-driven device needs to be fast and feasible in real-time, despite the existing limitations in terms of memory and processor capability. The same holds for the ability to analyze large ECG recordings collected over one or more days. Therefore, the main goal of this study is to produce a fast robust QRS detector that suits battery-driven applications and continuous 24/7 ECG monitoring, with theoretical justification for its parameters choice, tested over 11 large-standard datasets with different sampling frequencies, recording lengths, and noise. This study seeks to compare the various QRS detection methods against the developed QRS detection on standard databases. Furthermore, the theoretical basis of the well-known Pan and Tompkins algorithm [3] will be analyzed and evaluated against the proposed algorithm. The failures will be discussed, and the processing time of the proposed algorithm will be elaborated on.

## Materials and Methods

### Data Used

Several established ECG databases are available for evaluating QRS detection algorithms for ECG signals. As a sufficiently broad test scenario, 11 representative datasets published on PhysioNet [18] served for analyzing and comparing the proposed algorithm. These sets represent different subject groups and recording conditions, such as sampling rates (between 128 Hz and 1 kHz) and interferences. Lead I of every record is used without any exclusion. The corresponding reference R markers provided in the datasets acted as the benchmark.

### Training Set

**Figure 1:**
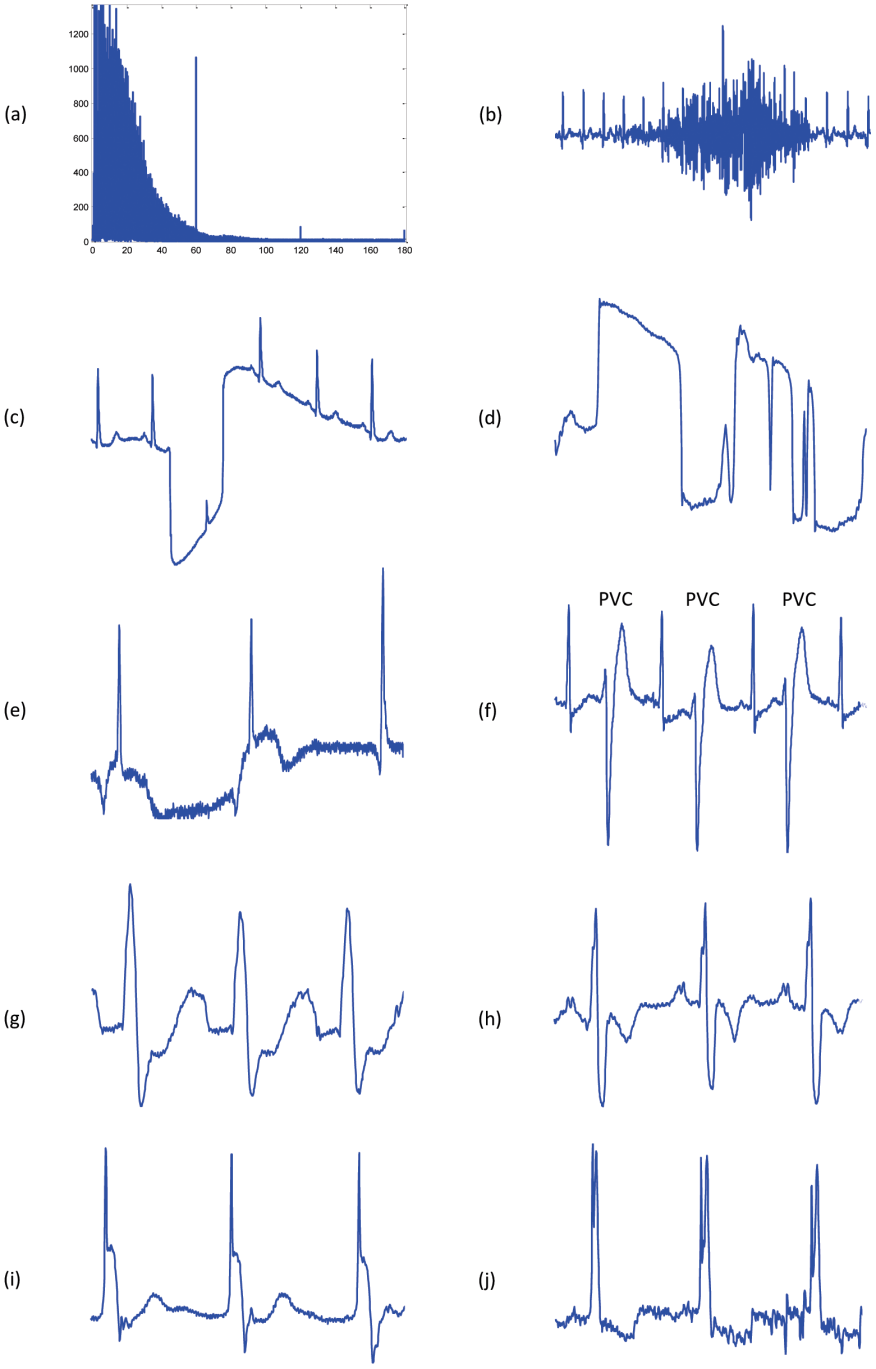
Challenges in detecting QRS in ECG signals. (a) Mains electricity noise: the spectrum illustrates peaks at the fundamental frequency of 60 Hz as well as the second and third harmonics at 120 Hz and 180 Hz, caused by stray magnetic fields causing the enclosure and accessories to vibrate. (b) High frequency noise caused by coughing. (c) Large movement of the chest. (d) Isolated QRS-like artifacts. (e) Nodal (junctional) escape beats affected by baseline wandering. (f) Premature ventricular contractions. (g) Left bundle branch block. (h) Right bundle branch block. (i) Paced beat. (j) Fusion of paced and normal beat.

The MIT-BIH Arrhythmia Database [5] is widely used to evaluate QRS detection algorithms. However, in this study, the database used for training as it includes different shapes of arrhythmic QRS complexes and noise. Most prominent were power-line interferences (60 Hz and its harmonics, see Fig. 1 (a)), which are known to be a major disturbance [6]. In addition, the design considered high-frequency noise, mostly originating from muscle activation (see Fig. 1 (b)), as well as low-frequency baseline fluctuations (see Fig. 1 (c, d, e)). Among the representative physiological events present in the datasets, special attention was paid to missing P waves (e.g., in junctional escape beats [7]; Fig. 1 (e)) and inverted (e.g., premature ventricular contractions, Fig. 1 (f)), notched (left bundle branch block, Fig. 1 (g)), as well as biphasic QRS complexes (right bundle branch block, Fig. 1 (h)). Finally, pacing-related phenomena were taken into account (see Fig. 1 (i, j)).

### Testing Set

Ten datasets were used for testing: the meta-dataset QT Database with 111,301 beats [8]; the T-Wave Alternans database with 19,003 beats, selected for its wide range of pathological conditions [9]; the Intracardiac Atrial Fibrillation database with 6,705 beats [10]; the ST Change database with 76,181 beats featuring stress ECGs [11]; the Supraventricular Arrhythmia database with 184,744 beats [12]; the Atrial Fibrillation Termination database with 7,618 beats [13]; the Fantasia database with 278,996 beats from relaxed healthy subjects [14]; the Noise Stress Test database with 26,370 beats recorded under noise conditions typical for clinical environments [15]; the St. Petersburg Institute of Cardiological Technics Arrhythmia database with 175,918 beats [7]; and the Normal Sinus Rhythm database with 183,092 beats [7]. In the Fantasia database, one record (‘f2y02’) was corrupted and was accordingly excluded. These benchmark datasets were selected for testing because of their representative character regarding pathological and typical ECG artifacts. Consequently, these were taken into account in testing the robustness of the proposed method.

## Methodology

**Figure 2:**
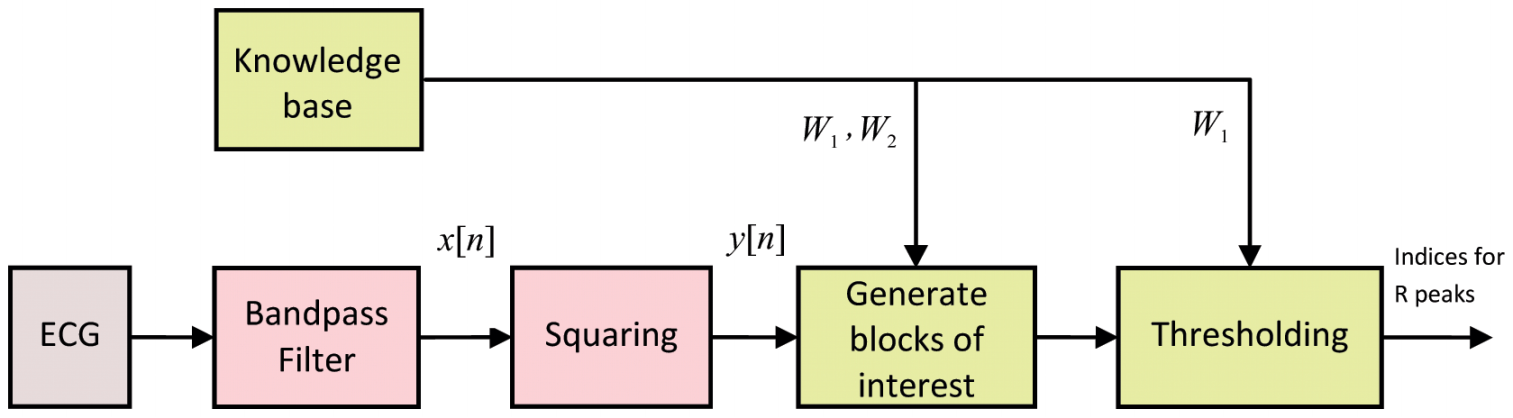
Flowchart of the knowledge-based QRS detection algorithm. The algorithm consists of three stages: pre-processing (bandpass filter and squaring), feature extraction (generating blocks of interest based on prior knowledge), and thresholding (based on prior knowledge).

In this section, a new, knowledge-based, numerically efficient, and robust algorithm is proposed to detect QRS complexes in ECG signals based on two event-related moving-average filters. The structure of the proposed algorithm is shown in Figure 2. It is clear that the knowledge base supports the decision making of both stages: generating blocks of interest and thresholding. It is expected that developing a detector that depends on prior knowledge of the ECG features will improve the overall performance and detection accuracy. Clifford et al. [16] provided a mini knowledge-base of the normal limits for the main events within the EGG, for a healthy male adult at 60 beats per minute (bpm), shown in Table 1.

**Table 1:** ECG features and their normal values in sinus rhythm.

Feature	Normal Value	Normal Limit	Normal duration for sampling frequency of 360 Hz
**P width**	110 ms	±20 ms	33–47 samples
**PQ/PR interval**	160 ms	±40 ms	43–72 samples
**QRS width**	100 ms	±20 ms	29–43 samples

The ECG features (P, QRS, and T waves) measured from a healthy male adult at a heart rate of 60 beats per minute (bpm). It is critical for the new developed algorithms to have an estimate for the event duration before processing the ECG signal. These durations play a role in determining the window size of the moving averages and threshold values.

The prior knowledge of the duration of the main events of the ECG signals can assist the feature extraction and support the decision making of the algorithm. For example, in this work, knowing that the QRS duration in a normal healthy subject varies from 29 to 43 samples, for a sampling frequency (SF) of 360 Hz, determines 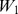 in generating blocks of interest and thresholding (cf. Figure 2). Similarly, the average heartbeat duration determines 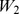 in generating blocks of interest. The average value for heartbeat duration is one second in healthy subjects, which means 360 samples (for a sampling frequency of 360 Hz). At this stage, 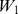 and 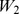 can be initialized by the prior knowledge that has been mentioned above. However, these durations vary from person to person. Therefore, the exact value for 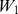 (QRS duration) and 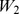 (one beat duration) will be determined after a brute force search, which will be discussed later in the parameter optimization section.

### Band-pass Filter

**Figure 3:**
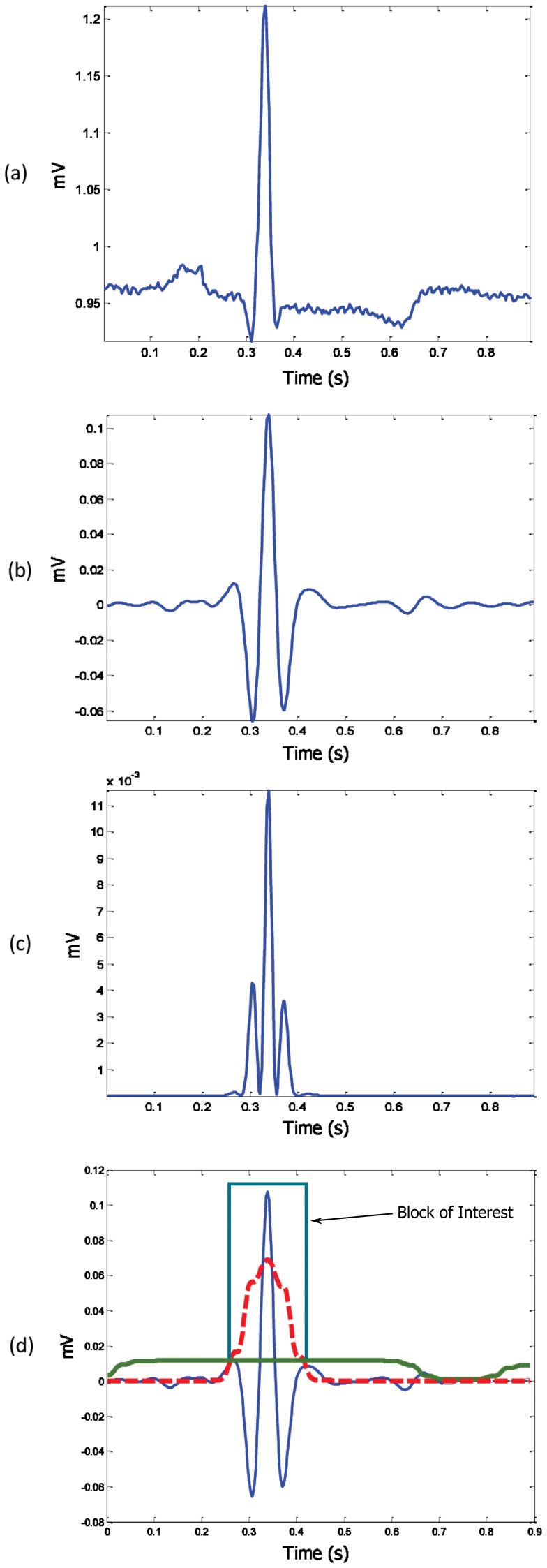
Demonstrating the effectiveness of using two moving averages to detect the QRS complex. (a) One beat ECG signal, (b) filtered one-beat ECG signal with Butterworth band-pass filter, (c) squaring the filtered signal, and (d) generating a block of interest after using two event-related moving averages: The dotted red line is the 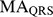, and the solid green line is the 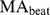. The R peak within the block of interest is then detected after the event-related threshold is applied.

Morphologies of normal and abnormal QRS complexes differ widely. The ECG signal is often corrupted by noise from many sources, which has been discussed. Therefore, band-pass filtering is an essential first step for nearly all QRS detection algorithms. The purpose of band-pass filtering is to remove the baseline wander and high frequencies that do not contribute to detecting QRS complexes. A band-pass filter is used, typically a bidirectional Butterworth implementation [17]. It offers good transition-band characteristics at low coefficient orders, which makes it efficient to implement [17]. Thakor et al. [18] and Chen and Chen [19] scored high accuracy using a third-order Butterworth filter with a passband of 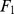
–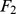 Hz to remove baseline wander and high frequencies, and to suppress the P and T waves and maximize the QRS area, where 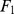 is the starting frequency and 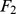 is the stopping frequency. The effect of the Butterworth filter can be seen in Figure 3 (b). However, rigorous optimization over the passband, to find the optimal frequency band, will be discussed in the parameter optimization section.

### Squaring Function

The signal is squared point by point, to enhance large values and boost high-frequency components, using the following equation:
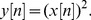
(1)The impact of the squaring is shown in Figure 3 (c).

### Generating Blocks of Interest

Blocks of interest are generated using two event-related moving averages. The first moving average 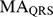 is used to extract the QRS features while the second-moving average 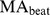 extracts the QRSs beat. Then, an event-related threshold is applied to the generated blocks to distinguish the blocks that contain R peaks from the blocks that include noise. The purpose of the QRS moving (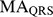) average is to smooth out multiple peaks corresponding to QRS complex intervals in order to emphasize and extract the QRS area:
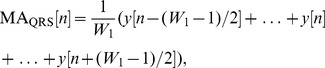
(2)where 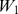 is the approximate duration of the QRS complex, rounded to the nearest odd integer, and 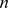 is the number of data points. Based on the knowledge-base analysis section, the QRS duration 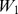 varies from 29 to 43 samples (for a sampling frequency of 360 Hz). Therefore, rigorous optimization to find the optimal 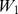 will be discussed in the parameter optimization section.

**Figure 4:**
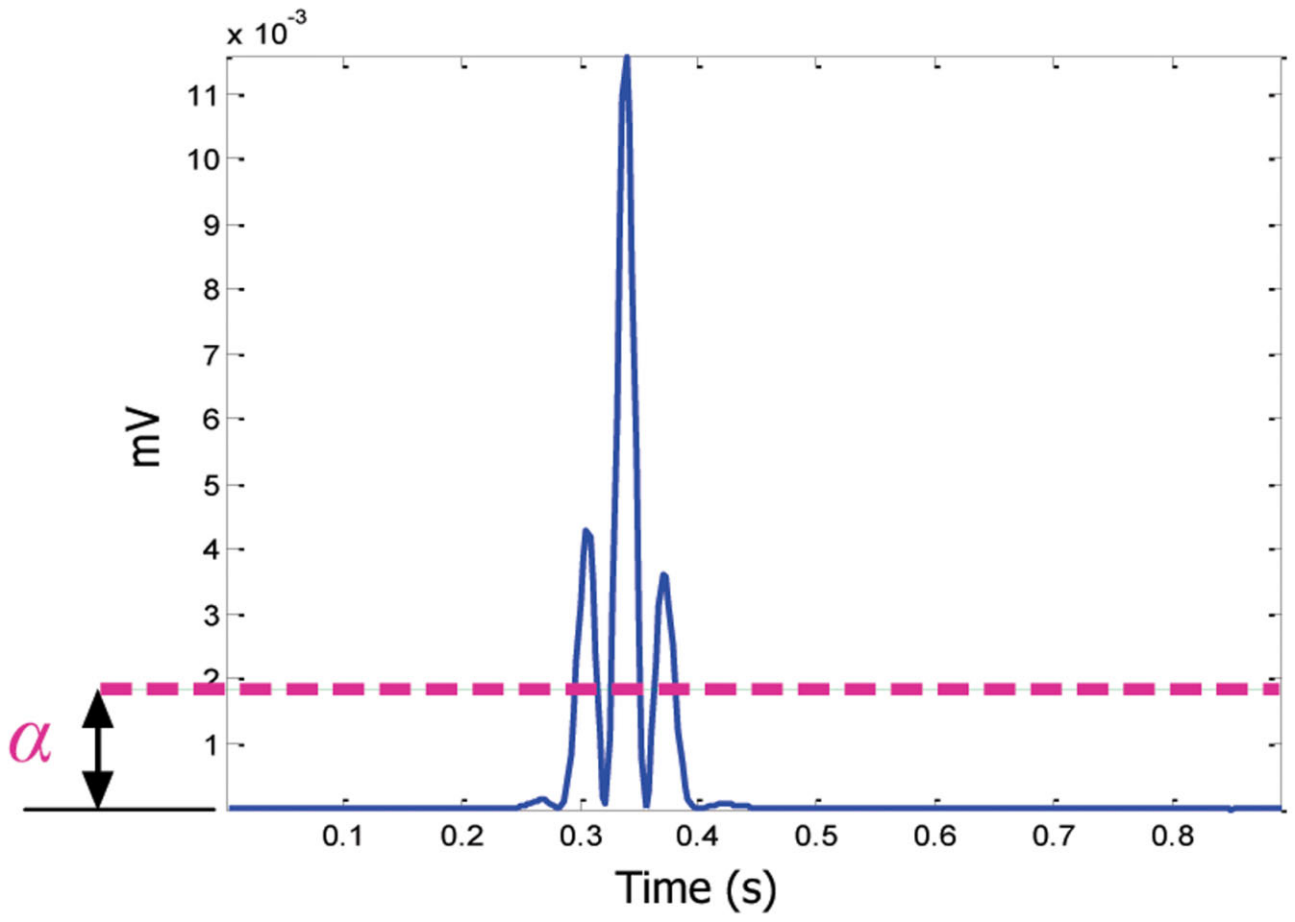
Demonstrating the statistical threshold. The squared one-beat ECG signal (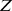), which is shown in Figure 3 (c), where the dashed line represents the offset caused by 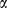.

The purpose of the one-beat moving average (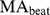) is similar to 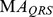 but emphasizes the QRSs beat to be used as a threshold for the first moving average (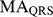):
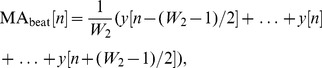
(3)where 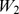 is the approximate duration of a heartbeat, rounded to the nearest odd integer, and 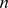 is the number of data points. Based on the knowledge-base analysis section, heartbeat duration 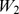 is about 360 samples (for a sampling frequency of 360 Hz); however, it varies from person to person. Rigorous optimization to find the optimal 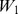 will be discussed in the parameter optimization section. The blocks of interest are generated based on the two moving averages discussed. In other words, applying the second-moving average 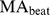 as a threshold to the first-moving average 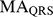 produces blocks of interest, as shown in Figure 3 (d). However, the use of 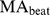 without an added offset reduces the detection accuracy because of its sensitivity to a low signal-to-noise ratio (SNR). The SNR defined the ratio of the mean signal of a region of interest to its standard deviation [20], which means if the statistical mean of the signal increases, the SNR increases. This leads to introducing an offset based on the statistical mean of the signal as
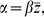
(4)where 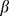 is the fraction of the 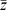 signal that needs to be removed, 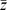 is the statistical mean of the squared ECG signal 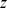, as illustrated in Figure 4, and 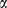 is an offset for the threshold 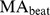 signal. Thus, 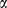 refers to the *offset*, while 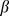 refers to the *offset fraction*.

**Figure 5:**
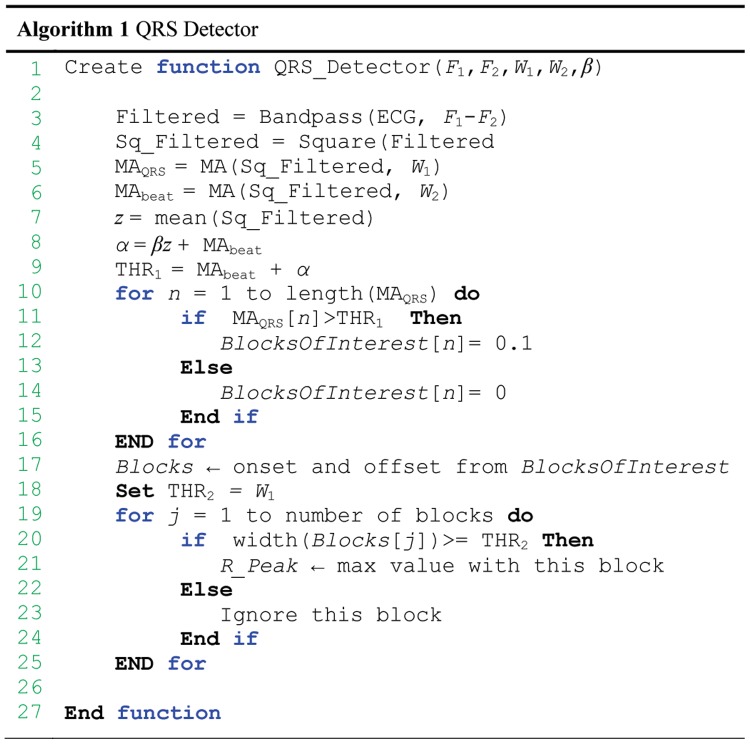
Pseudocode for the knowledge-based QRS detector function. The function has five inputs: 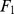, 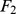, 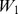, 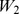, and 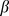. The band-pass filter will be determined by the frequency band 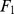
–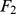 Hz, while 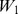 and 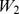 are the window sizes of the two moving averages 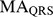 and 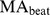, respectively. However, 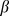 is used to calculate the statistical threshold 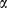.

In short, to increase the accuracy of detecting QRS complexes in noisy ECG signals, the dynamic threshold value 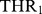 is calculated by offsetting the 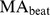 signal with 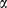, as follows:
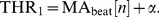
(5)The blocks of interest are then generated by comparing the 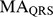 signal with 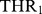. If a block is higher than 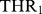, it is classified as a block of interest containing ECG features (P, QRS, or T) and noise; otherwise, as shown in lines 10–16 in Figure 5. By this stage, blocks of interest have been generated, 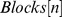. Therefore, the next step is to reject the blocks that result from noise. The rejection should be related to the anticipated block width.

### Thresholding

Here, the undesired blocks are rejected by using the new 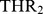 threshold to reject the blocks that contain P and T waves and noise. By applying the 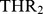 threshold, the accepted blocks contain only QRS complexes:
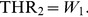
(6)As discussed, the threshold 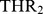 equals 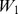, which corresponds to the anticipated healthy QRS width. If the block width equals the window size 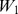, then the block contains a QRS complex. However, the QRS duration varies in arrhythmia ECG signal durations. Therefore, the condition is set to capture both average (healthy beats) and wide (arrhythmia beats) QRS complex durations. Therefore, if a block width is greater than or equal to 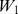, it is classified as a QRS complex. If not, the block is classified as a P wave, T wave or noise.

### Detecting R Peaks

The last stage is finding the maximum absolute value within each block, the R peak.

### Parameters Optimization

**Figure 6:**
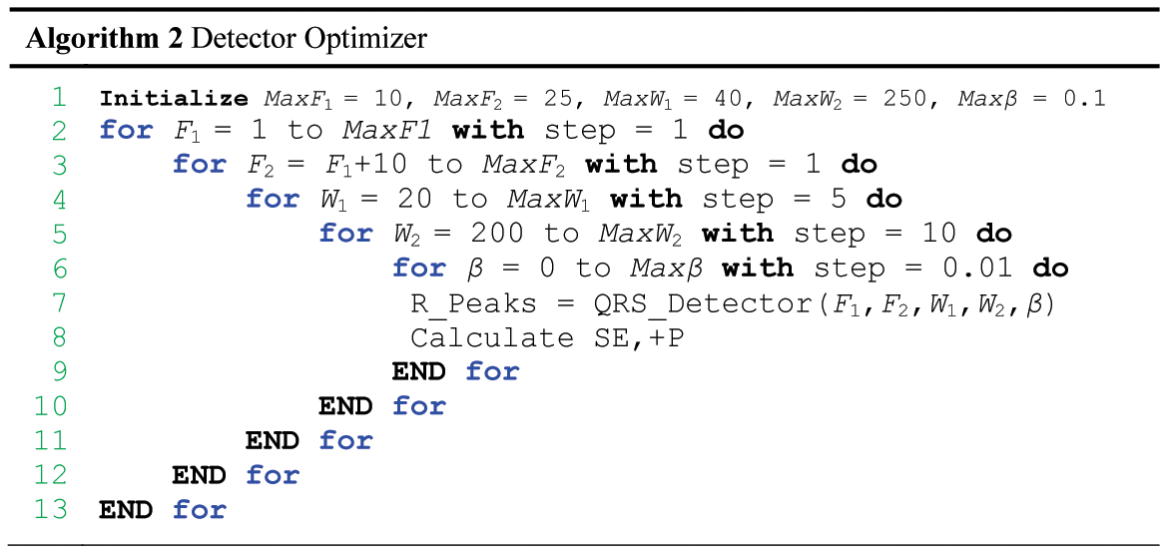
Pseudocode for the brute-force optimizer. The optimizer is initialized with 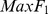 = 10 Hz, 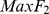 = 25 Hz, 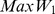 = 40 samples, 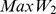 = 250 samples, and 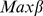 = 0.1. Systematically, this exhaustive search enumerates all possible combinations for the solution and checks whether each combination provides an optimal detector based on SE and +P.

The function of the QRS detector, which is presented in Figure 5, has five inputs: the frequency band (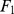
–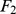), event-related durations 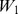 and 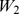, and the offset fraction (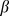). Any change in these parameters affects the overall performance of the proposed algorithm. These parameters are interrelated and cannot be optimized in isolation. A rigorous optimization, brute-force search based on the knowledge-base information, over all parameters, is conducted, as shown in Figure 6. It is time-consuming, as the complexity of the algorithm is ((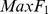-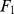) (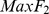-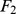) (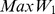-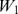) (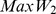-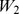) 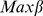), but it is required before making any claims. The MIT-BIH Arrhythmia Database was used for training and optimization.

## Results

The QRS detection algorithm is typically run using two statistical measures: sensitivity (SE) and positive predictivity (+P); whereas 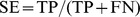 and 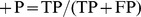. Here, TP is the number of true positives (QRS complexes detected as QRS complexes), FN is the number of false negatives (QRS complexes have not been detected as QRS complexes), and FP is the number of false positives (non-QRS complexes detected as QRS complexes). The SE reports the percentage of true beats that were correctly detected by the algorithm. The +P reports the percentage of beat detections that were true beats.

### Training Results

Figure 6 shows that the optimizations of the beat detector's spectral window for lower frequency varied from 1–10 Hz, with the higher frequency up to 26 Hz. All combinations of the frequency band 1–26 Hz have been explored to include all frequency bands that have been recommended in the literature such as 8–20 Hz [21], 5–15 Hz [18], [19], and 5–11 Hz [3]. The window size of the 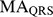 (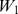) ranged from 55 to 111 ms, whereas the window size of the 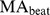 (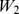) changed from 555 ms to 694 ms as discussed in the knowledge-base analysis section. However, the offset was tested over the range 0–10% of the mean value of the squared filtered ECG signal. The database used in the optimization process is the MIT-BIH Arrhythmia Database because it contains abnormal rhythms, different QRS morphologies, and low SNR signals, as described in the Challenges in the ECG section. The total number of beats in the MIT-BIH Arrhythmia Database is 109,984, and there are 48 records. As discussed, several publications have listed the use of all files in the database, excluding just the paced patients, segments, and certain beats. However, in the optimization process all records have been used without excluding any beat. After the rigorous optimization, all parameter combinations were sorted in descending order according to the overall accuracy, as shown in Table 2; thus, the first combination provides the optimal solution. The highest overall-accuracy score is 99.83% (cf. Table 2); therefore, the optimal frequency range for QRS detection in the MIT-BIH Arrhythmia Database is 8–20 Hz, as proposed by Benitez et al. [21]. Moreover, the optimal values for the moving averages and offset are 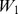 = 97 ms (35 samples for SF = 360 Hz) and 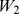 = 611 ms (220 samples for SF = 360 Hz), and 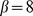. (Investigators do not have to think about the optimization as it is already done here for them; all they need to do is simply implement the proposed algorithm with these optimal parameters.)

**Table 2:** A rigorous optimization of all parameters of the algorithm: frequency band, *W*_1_, *W*_2_, and the offset fraction *β*.

Combination	Frequency Band	*W*_1_ (samples)	*W*_2_ (samples)	Offset (%)	SE (%)	+P (%)	Overall Accuracy (%)
1	8–20 Hz	35	220	8	99.78	99.87	99.83
2	8–19 Hz	40	220	10	99.76	99.89	99.83
3	9–19 Hz	40	220	9	99.77	99.89	99.83
4	8–20 Hz	40	220	8	99.79	99.87	99.83
5	8–20 Hz	35	220	9	99.77	99.89	99.83
6	8–19 Hz	35	220	9	99.76	99.89	99.83
7	8–21 Hz	40	220	8	99.79	99.87	99.83
8	8–21 Hz	40	230	9	99.77	99.89	99.83
9	9–19 Hz	35	230	9	99.76	99.89	99.83
10	8–21 Hz	35	220	9	99.77	99.88	99.82
11	8–21 Hz	35	210	9	99.78	99.87	99.82
12	8–18 Hz	40	220	10	99.75	99.90	99.82
13	8–21 Hz	40	220	9	99.77	99.88	99.82
14	8–18 Hz	35	220	9	99.76	99.89	99.82
15	8–21 Hz	35	220	8	99.78	99.86	99.82
16	8–22 Hz	35	220	9	99.77	99.88	99.82
17	8–22 Hz	35	220	10	99.75	99.89	99.82
**.**	**.**	**.**	**.**	**.**	**.**	**.**	**.**
**.**	**.**	**.**	**.**	**.**	**.**	**.**	**.**
37947	1–26 Hz	20	200	7	33.713	93.869	63.791
37948	1–26 Hz	20	200	8	33.263	93.907	63.585
37949	1–26 Hz	20	200	9	32.803	93.962	63.383
37950	1–26 Hz	20	200	10	32.371	94.127	63.249

All possible combinations of parameters (37,950 iterations) have been investigated and sorted into descending order according to their overall accuracy. The database used is the MIT-BIH Arrhythmia Database. The overall accuracy is the average value of SE and +P.

### Testing Results

Now, an optimal QRS detector is accomplished over the MIT-BIH Arrhythmia Database. Then, we can test this detector on other datasets straight out of the box without any tuning. In other words, the algorithm's parameters (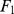, 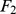, 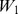, 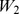, and 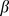) do not need to be trained in real-world application for every subject. The parameters are optimized on a large training set (MIT-BIH data set); thus, the robustness of the algorithm can be examined against different databases with different sampling frequencies and the ECG signals collected by different doctors in dissimilar conditions. Table 3 shows the performance of the QRS detection algorithm on 11 databases. In addition, the performances are summarized across these databases and compared to other reported results. Because the algorithm has not been re-tuned over any databases, the results are promising, and the algorithm can detect R peaks over different databases, sampling frequencies, types of arrhythmias, and types of noise. The number of beats used to calculate these performance parameters is indicated in the second column in Table 3. Hamilton and Tompkins implemented their QRS detection algorithm in 1986. They scored 99.69% SE and 99.77% +P over 109,267 beats from the MIT-BIH Database (cf. Table 3). When Arzeno et al. [21] applied the Hamilton-Tompkins algorithm over a slightly larger number of beats, 109,504 beats, the detector performance decreased slightly, scoring a SE of 99.68% and a +P of 99.63%.

**Table 3:** QRS Detection performance comparison on 11 databases (Lead I).

Database	No. of beats	No. of beats	SE (%)	+P (%)
**MITDB**	This work	109985	99.78	99.87
	Pan and Tompkins	109985	90.95	99.56
	Chouhan [22], [23]	44677	87.90	97.60
	Improved Chouhan [22], [23]	44677	97.5	99.90
	Adnane et al. [24]	109487	99.77	99.64
	Ghaffari et al. [25]	109837	99.91	99.72
	Benitez et al. [21]	109257	99.13	99.31
	Modified Benitez et al. [21]	109517	99.29	99.24
	Hamilton and Tompkins [21]	109504	99.68	99.63
	Modified Hamilton-Tompkins [21]	109436	99.57	99.58
	Second derivative of Hamilton-Tompkins [21]	108228	98.08	99.18
	Zheng and Wu [26]	N/R	98.68	99.59
	Fard et al. (HCW) [27]	24000	99.79	99.89
	Fard et al. (CFBSW) [27]	24000	99.29	99.89
	Fard et al. (CNW) [27]	24000	99.49	99.29
	Darrington [28]	109487	99.06	99.20
	Chen et al. [29]	102654	99.55	99.49
	Martinez et al. [30]	109428	99.80	99.86
	Hamilton [31]	N/R	99.80	99.80
	Lee et al. [32]	109481	99.69	99.88
	Afonso et al. [33]	90909	99.59	99.56
	Li et al. [34]	104182	99.89	99.94
	Hamilton and Tompkins [35]	109267	99.69	99.77
**QTDB**	This work	111201	99.99	99.67
	Pan and Tompkins	111201	97.99	99.05
	Martinez et al. [30]	86892	99.92	99.88
	Aristotle [30]	86892	97.20	99.46
**NSTDB**	This work	26370	95.39	90.25
	Pan and Tompkins	26370	74.46	93.67
	Benitez et al. [36]	N/A	93.48	90.60
**TWADB**	This work	19003	98.88	99.12
	Pan and Tompkins	19003	88.32	94.04
**STDB**	This work	76181	99.92	99.70
	Pan and Tompkins	76181	91.78	98.95
**SVDB**	This work	184744	99.96	99.80
	Pan and Tompkins	184744	99.27	98.34
**IAFDB**	This work	6705	99.59	94.11
	Pan and Tompkins	6705	64.21	99.01
**NSRDB**	This work	183092	99.99	99.96
	Pan and Tompkins	183092	99.91	99.97
**AFTDB**	This work	7618	99.72	99.74
	Pan and Tompkins	7618	96.62	99.4
**FANTASIADB**	This work	278996	99.98	99.87
	Pan and Tompkins	278996	89.16	99.89
**INCARTDB**	This work	175918	99.03	97.09
	Pan and Tompkins	175918	49.75	97.49

(N/R: NOT REPORTED).

Li et al. [34] scored higher performance, a sensitivity of 99.89% and a specificity of 99.94%, than the proposed algorithm. This is because Li et al. excluded files 214 and 215 from the MIT-BIH Database, and therefore, the algorithm is not superior in terms of performance. However, their algorithm was based on wavelets feature extraction and singularity for classification, which is considered numerically inefficient. Moreover, the algorithm developed by Ghaffari et al. [25] scored a sensitivity of 99.91% and a specificity of 99.72% over 109,837 beats (not all beats); their algorithm was based on wavelets feature extraction and thresholds for classification, which is also considered numerically inefficient. Conversely, the proposed knowledge-based algorithm presents a clear advantage over the previously reported algorithms in terms of performance (large number of databases) and numerical efficiency. This was clear with the MIT-BIH Arrhythmia Database, as discussed above. In addition, the QTDB where the detector scored an SE of 99.67% and a +P of 100%, over 111,193 beats, without excluding any beats as Martinez et al. [30] and Aristotle [30] did. Furthermore, the overall performance of the detector on the NSTDB was higher than Benitez et al. [36], with clear mentioning of the number of beats used, specifically 26,370 beats.

## Discussion

After the description of the detector and its results on different datasets, perhaps further elaboration on the detectors performance is required. However, comparing the performance of the proposed algorithm with previously published algorithms is difficult. This is because the algorithms are not tested on the same data, in particular the same beats. By excluding the number of beats and/or certain records, the performance of any detector will score higher detection rates. Here are a few examples to clarify the idea:

- Xue et al. [37] reported sensitivities of 99.84% and 99.09% and positive predictivity of 99.61% and 98.59% based on just two records, 105 and 108 from the MIT-BIH Arrhythmia Database.
- Wavelet transforms were used for QRS detection by Li et al. [34]. They reported 0.15% false detections based on 46 files from the MIT-BIH Arrhythmia Database, excluding files 214 and 215.
- Moraes et al. [38] logically combined two different algorithms working in parallel, the first adopted from the work of Englese and Zeelenberg [39], the second based on Pan and Tompkins [3] and Ligtenberg and Kunt [40]. Moraes et al. reported sensitivity of 99.22% and specificity of 99.73% after having excluded records of patients with pacemakers. However, they also excluded recordings 108, 200, 201, and 203, from the MIT-BIH Arrhythmia Database.
- Continuous spline wavelet transform using local maxima of the continuous wavelet transform at different scales have been used by Alvarado et al. [41]. They reported sensitivity of 99.87% and positive predictivity of 99.82% after using just nine files out of 48 files from MIT-BIH Arrhythmia Database.
- Zhang et al. [42] used the continuous wavelet transform, followed by fixed thresholds. They reported accuracy of 99.5% after using just eight files out of 48 files from MIT-BIH Arrhythmia Database.

Most of the proposed algorithms were tested on one dataset, the MIT-BIH Arrhythmia Database. The authors exclude some records from the database to improve the overall accuracy. Here is an example based on the proposed detector: If records 108 and 207 are excluded from this study, the proposed detector scores SE of 99.9% and +P of 99.95%, which does not reflect the real performance of the algorithm. Therefore, the author urges readers, researchers, and biomedical-signal-analysis community of using the standard databases with excluding any record or beat. Now, after the misleading conclusions based on data elimination have been discussed, the performance of the proposed detector can be discussed technically. The main technical aspects of any QRS detector are frequency-band choice, window-size and threshold choices, failure, and processing time.

### Implementation Steps

In general, the Pan and Tompkins algorithm is more complex compared to the proposed algorithm, and thus has more implementation steps, as shown in Table 4. The Pan and Tompkins algorithm requires a resampling step for any ECG signal not sampled at 200 Hz. Its filters are designed for 200 Hz, so performance will be degraded at other sampling frequencies. Moreover, as the Pan and Tompkins algorithm is amplitude dependent, subtraction of the statistical mean of the ECG signals is also required. It also imposes a differentiation step to emphasize the QRS complex slope information. Furthermore, the thresholding step is complicated (really this word, not just a phrase that contains it) compared to that of the proposed algorithm. The thresholding code of the Pan and Tompkins algorithm is taken from DigiScope software [43].

**Table 4:** Comparison between the proposed QRS detector and the Pan and Tompkins algorithm.

Step	Proposed Detector	Pan and Tompkins [3]
Resampling	N/A	Resample *ECG* to 200 Hz
Mean Subtraction	N/A	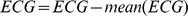
Frequency Band	*x* = bandpass(*ECG*, 8–20 Hz)	*x* = bandpass(*ECG*, 5–15 Hz)
Differentiation	N/A	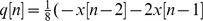
		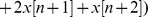
Squaring	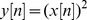	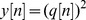
Integration	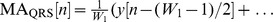	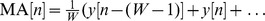
	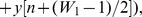	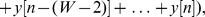
	where *W*_1_ = 97 ms	where *W* = 150 ms
	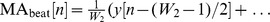	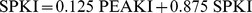
	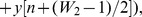	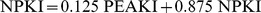
	where *W*_2_ = 611 ms	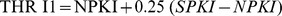
	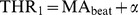	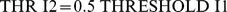
Thresholds	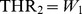	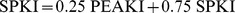
		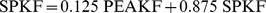
		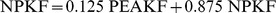
		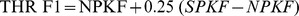
		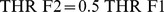
		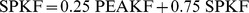
		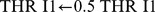
		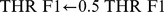
		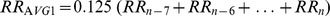
		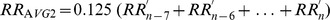
Adjusting thresholds	N/A	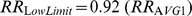
		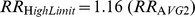
		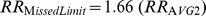
		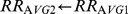

Here, N/A means NOT APPLIED.

### Frequency-Band Choice

In the literature, the QRS frequency band has been used without actually identifying the optimum QRS frequency range for the detection of the QRS complexes. Different researchers used different passbands; for example, Thakor et al. [18] proposed an estimate of QRS complex spectra and suggested that the passband that maximizes the QRS energy is approximately 5–15 Hz. Pan and Tompkins [3] used cascaded low-pass and high-pass filters to achieve a passband of about 5–11 Hz. Li et al. [34] used a quadratic spline wavelet with compact support and one vanishing moment. They concluded that most QRS complex energies are at the scale of 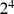; that is, the Fourier transform frequency range lies between 4 and the 13.5 Hz. Sahambi et al. [44] used the first derivative of a Gaussian smoothing wavelet and found that most QRS complex energies are at the scales of 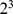 and 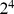, with corresponding frequency ranges between 4.1 Hz and 33.1 Hz. Benitez et al. [36] developed a QRS detection algorithm using the properties of the Hilbert transform with band stop frequencies at 8 and 20 Hz in order to remove muscular noise and maximize the QRS complex, respectively. Moraes et al. [38] combined two improved QRS detectors using a band-pass filter between 9 and 30 Hz. Chen and Chen [19] introduced a QRS detection algorithm based on real-time moving averages and assumed the QRS frequencies were concentrated at approximately 5–15 Hz. Mahmoodabadi et al. [45] used Daubechies2 to detect QRS complex using scales of 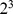
–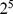, which covers the frequency range 2.2–33.3 Hz.

**Figure 7:**
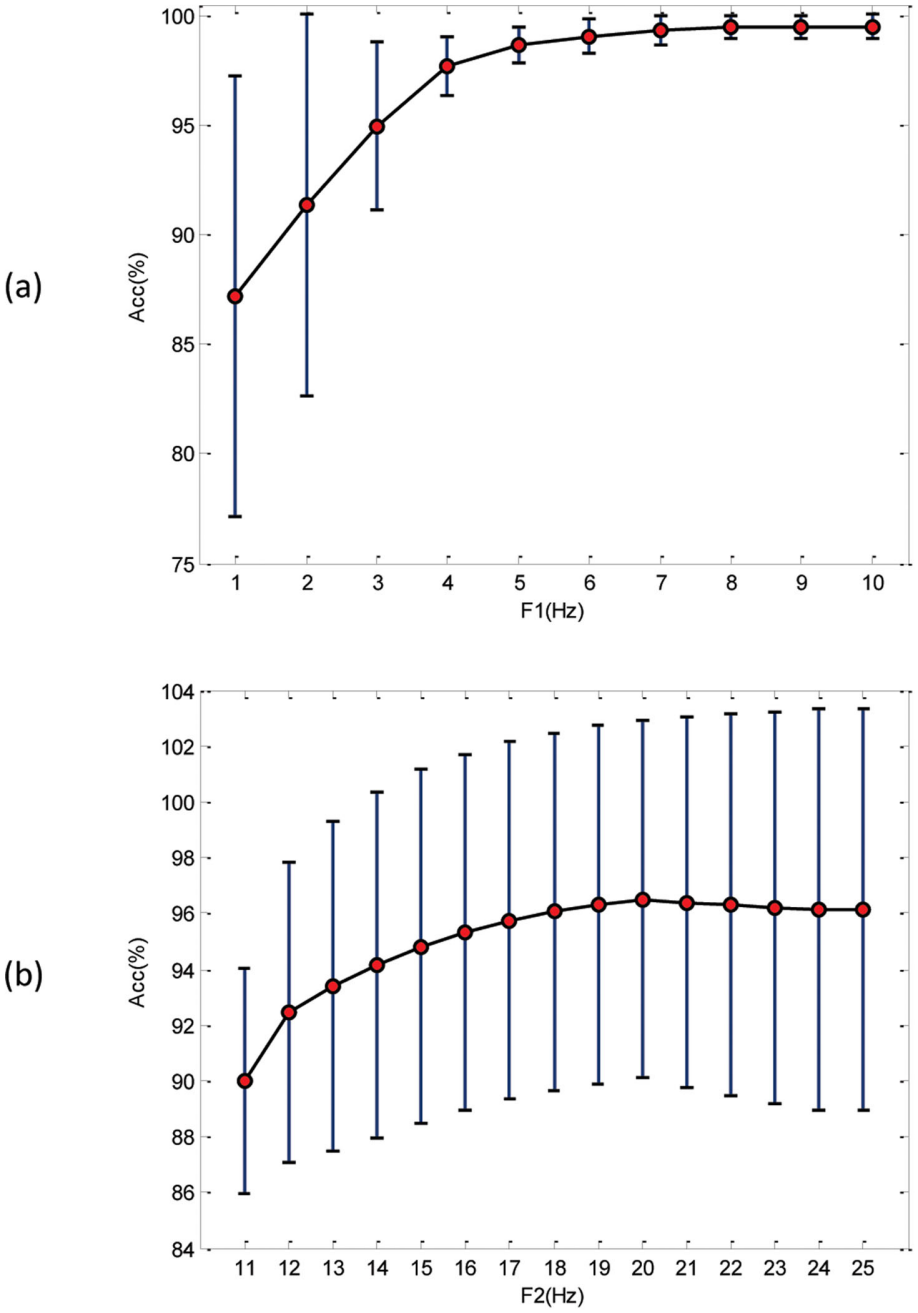
Influence of frequency bands on the overall accuracy based on brute-force optimization. (a) Frequency band starts at value within 1–10 Hz. (b) Frequency band stops at value within 11–25 Hz, where the circle is the statistical mean, and the bar is the standard deviation.

Most of these authors evaluated their algorithms using the MIT-BIH Arrhythmia Database and determined the frequency bands experimentally, without justifying their choice. Thus, an optimal frequency band for detecting QRS complexes is proposed based on rigorous brute-force optimization, which is 8–20 Hz, as elaborated in the parameter optimization section. This result confirms the findings of Elgendi et al. [46] that 8–20 Hz optimizes the QRS detection. Moreover, Figure 7 shows the influence of a certain frequency band on the overall accuracy. It is clear that 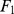 scores consistent results above 5 Hz, as shown in Figure 7(a). Thus, in designing a band-pass filter, the starting frequency should lie within 5–10 Hz. Regarding the stopping frequency, 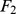, perhaps the optimal choice is 20 Hz, which has the highest average and lowest standard deviation; 19 and 21 Hz can still provide relatively high accuracy.

### Window-Size and Threshold Choices

**Figure 8:**
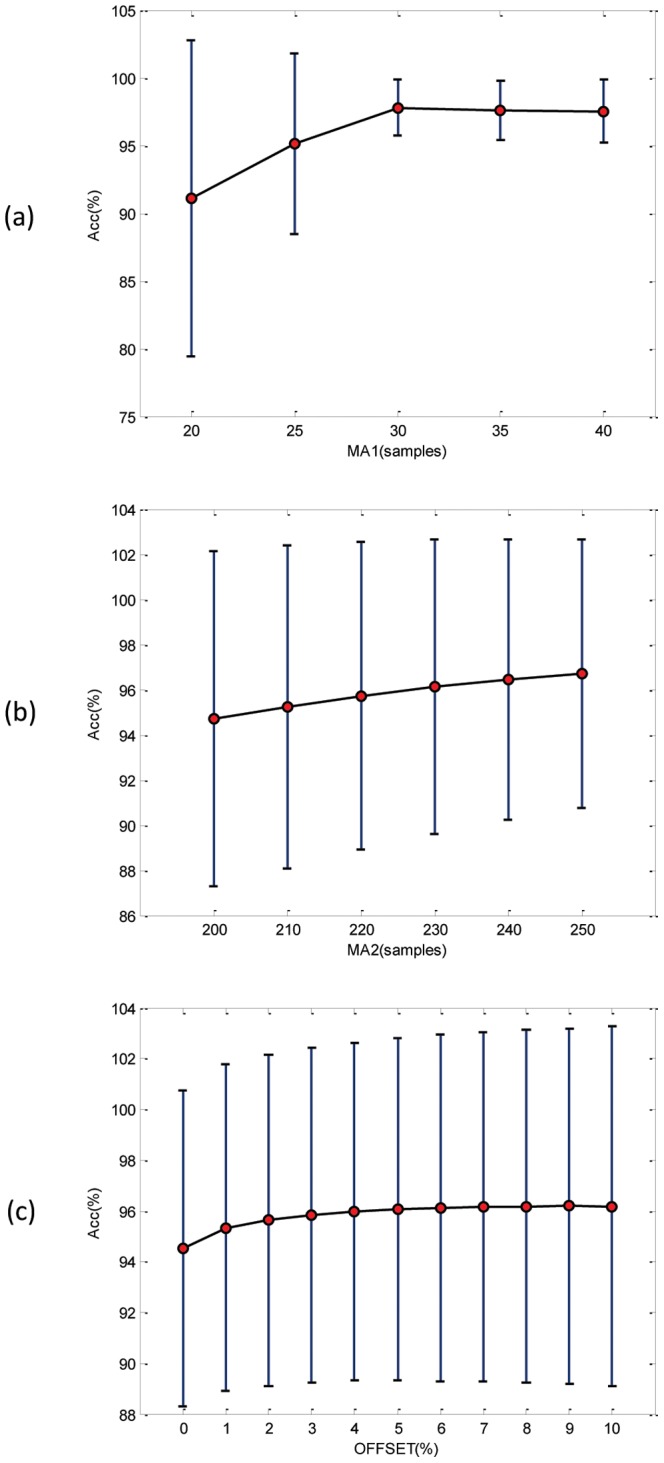
Influence of window sizes and offset on the overall accuracy based on brute-force optimization. (a) The window size of the 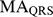 varies from 20 to 40 samples, for SF = 360 Hz. (b) The window size of the 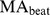 varies from 200 to 250 samples for SF = 360 Hz. (c) The offset fraction 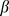 varies from 0 to 10%, where the circle is the statistical mean, and the bar is the standard deviation.

The window size is an important factor in detection; it should reflect the duration of the QRS complex, which is an individual characteristic that further depends on the heart rate, and thus is hard to predict. Researchers generally use a fixed window size for the moving average that demarcates the QRS complex; for example, Pan and Tompkins [3] used a fixed window size of 30 (which is 150 ms). However, their adaptive thresholds were based on the eight most recent beats [3]. The disadvantages of their algorithm are the window size is determined empirically and thresholds depend on the accuracy of the heart rate determined in the previous segment. A domino effect of errors will occur. Therefore, a new solution is needed that does not depend on the recent heart rate. The proposed method uses a predefined but on average perfect constant window size by searching for the optimized window sizes for the QRS and heartbeat durations. However, the algorithm shares some steps with Pan and Tompkins algorithm. A comparison is presented in Table 4 to show the main differences and the novelty of the proposed methodology, which is the optimized knowledge-base consideration. In addition to efficiency, the author aimed at reducing the complexity of detection methods. Therefore, the proposed method uses a predefined but on average optimal constant window size (see Table 4) to demarcate the QRS complex. The second moving average filter was implemented to eliminate the multiple static thresholds by demarcating each heartbeat, which works as a data-driven threshold for the first moving average 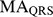. Thus, the proposed detector overcomes the unjustified parameters value and the use of fixed thresholds. Figure 8 shows the influence of the window sizes of the moving averages and offset on the overall accuracy. It is clear that the optimal window size 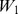 for detecting QRS can be 30, 35, or 40 samples (for SF = 360 Hz). The optimal window size 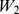 for demarcating a heartbeat was hard to determine, as it perhaps can be 220, 230, 240, or 250 samples (for SF = 360 Hz). The optimal offset fraction 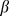 varies from 2 to 10% (cf. Figure 8 (c)). However, the optimal combination based on the brute-force search was 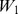 = 35 samples 97 ms, 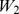 = 220 samples 611 ms, and the offset fraction was 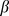 = 0.8, as shown in Table 2. Combinations 2 to 17, in Table 2, provide relatively high accuracy as well.

### Instances of Failure

**Figure 9:**
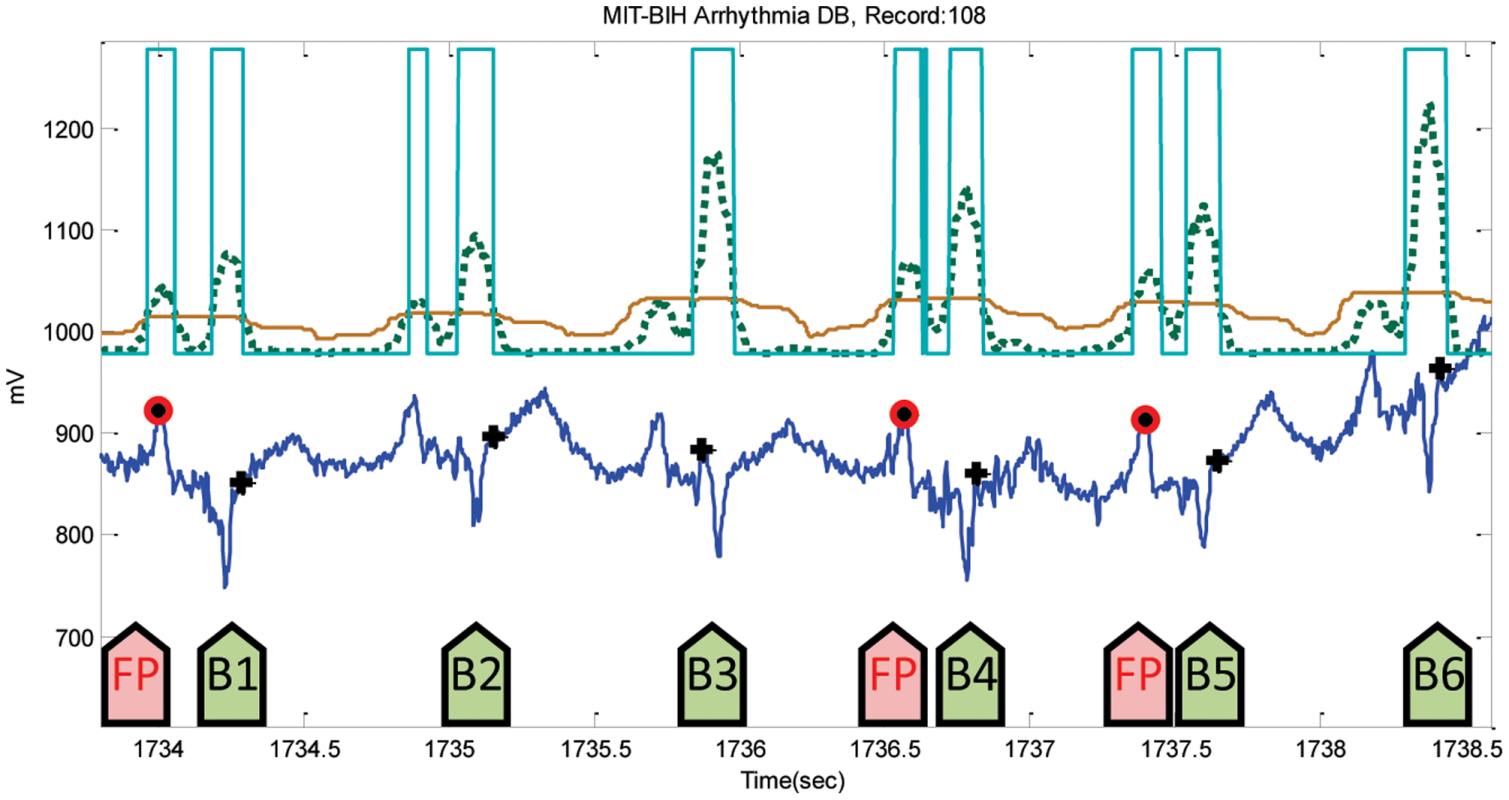
Noisy reversed-polarity QRS complexes in Record 108. The dotted line is the first moving average 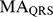, and the solid line is the second moving average 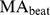. The green arrows point to successful detection, while the pink arrows point to failures. Here, the black plus sign represents successful detection produced by the proposed algorithm, where the red circle represents FP.

**Figure 10:**
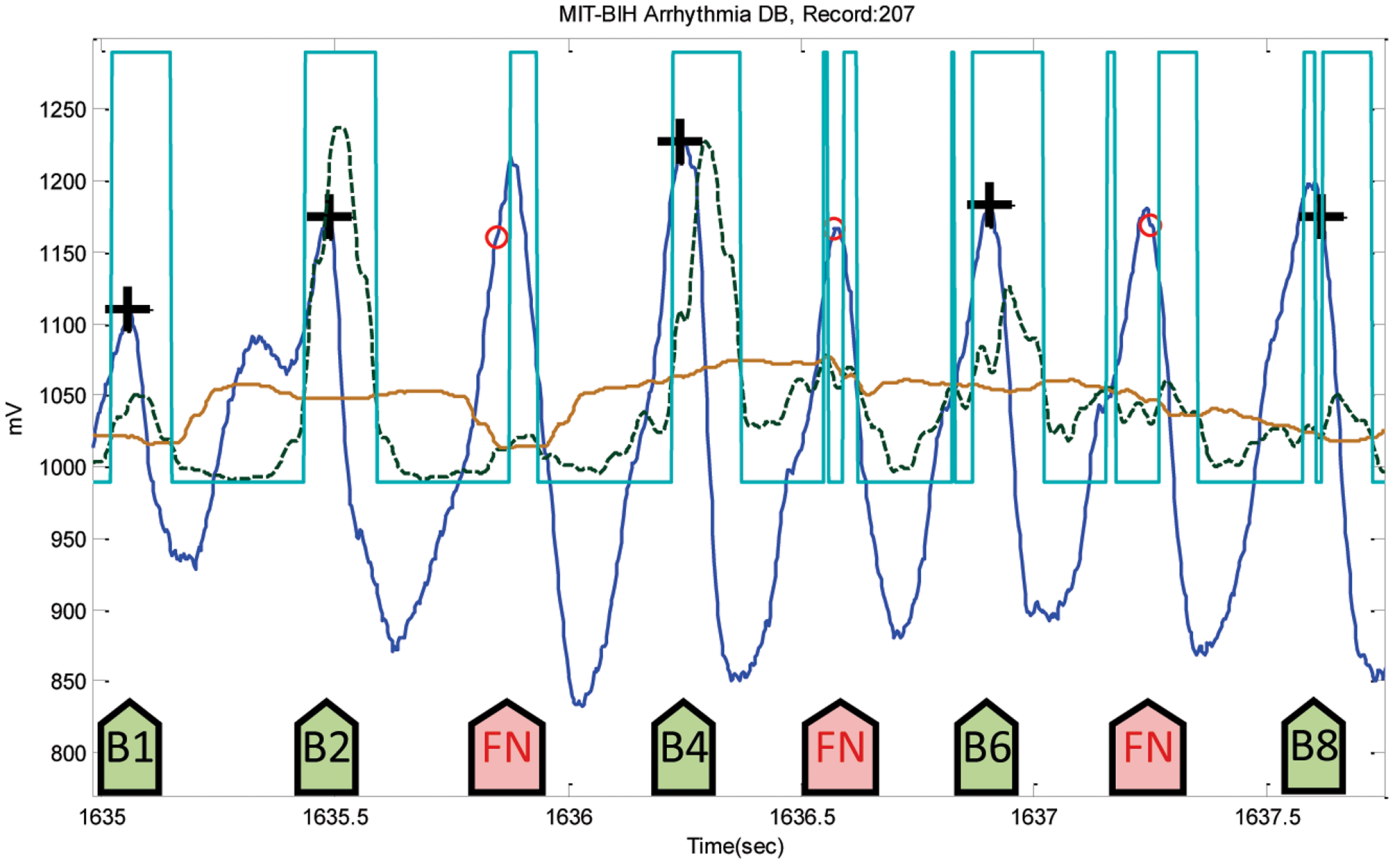
Ventricular flutters in Record 207-MITDB. The dotted line is the first moving average 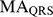, and the solid line is the second moving average 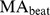. The green arrows point to successful detection, while the pink arrows point to failures. Here, the black plus sign represents successful detection produced by the proposed algorithm, where the red circle represents FN.

After the training phase, which is discussed in the parameters optimization section, the parameters of the developed QRS algorithm were optimized over the MIT-BIH Arrhythmia Database. The optimized algorithm will be tested on all other databases without any tuning. The algorithm has been used straight out of the box and has not been re-tuned over any databases. In the testing phase, usually algorithms fail at specific instances within the ECG recordings, which are considered either false positives (FPs) or false negatives (FNs). These instances of failure will be discussed over all databases, including the database used in training. The proposed algorithm incurred a total of 124 FPs and a total of 247 FNs over the MIT-BIH Arrhythmia Database. The noisy reversed QRS polarities caused the highest number of FPs in Record 108, as shown in Figure 9, while Record 207 scored the highest number of FNs, precisely 198 FNs, because of the ventricular flutters (cf. Figure 10). In Figure 9, the two moving averages succeeded in generating blocks of interest that demarcated all QRS complexes, but also demarcated the wide P waves, causing FPs before B1, B4, and B5 shown in Figure 9; and threshold 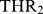 could not help in rejecting them. On the other hand, the moving averages could not generate blocks of interest due to the fast rhythm as B3, B5 and B7 show in Figure 10.

**Figure 11:**
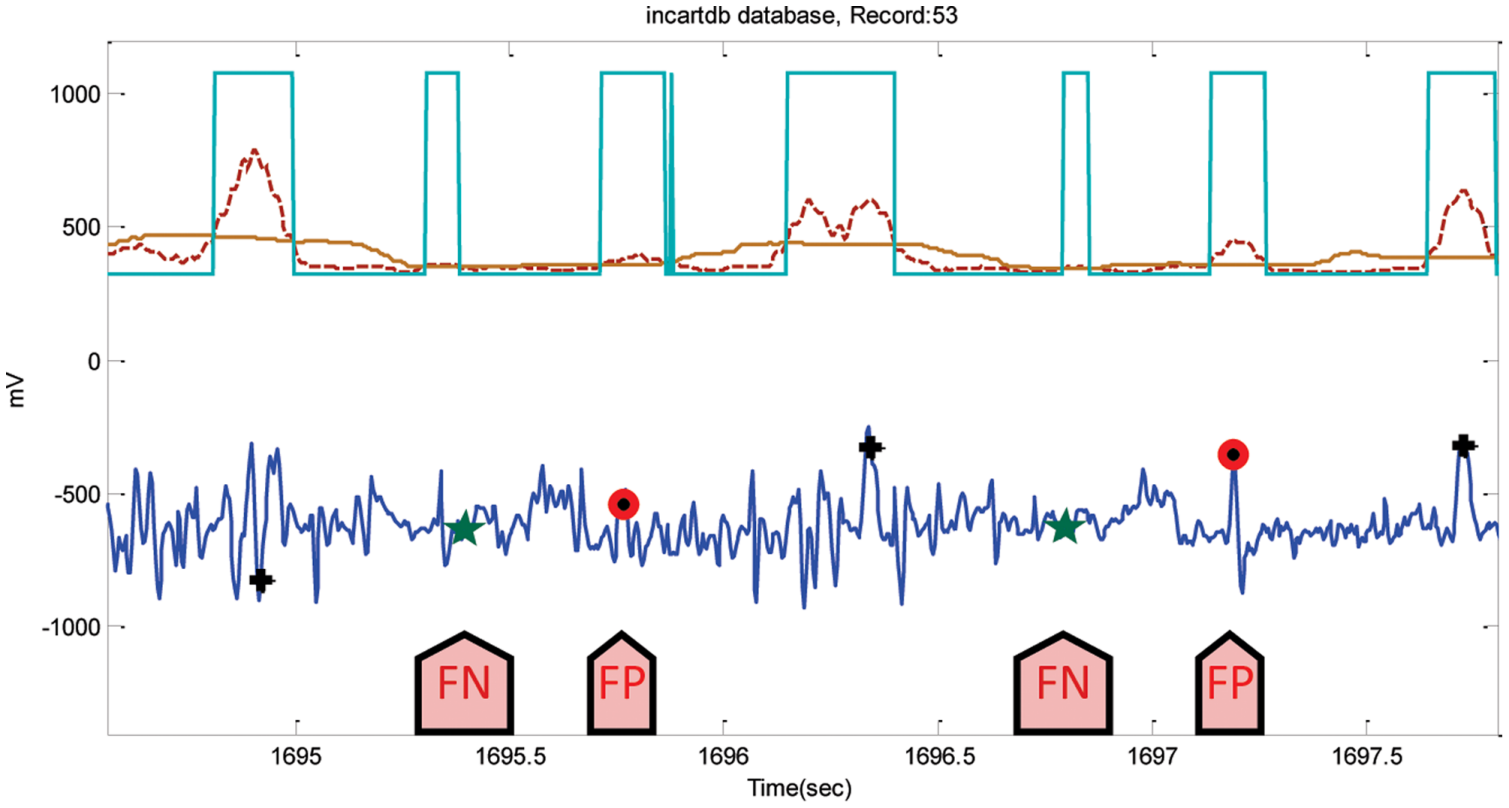
Noisy ECG signals in Record 53-INCARTDB. The dotted line is the first moving average, 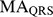, and the solid line is the second moving average 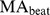. The arrows point to FNs and FPs. Here, the black plus sign represents successful detection produced by the proposed algorithm, where the red circle represents FP, and the green star represents FN.

**Figure 12:**
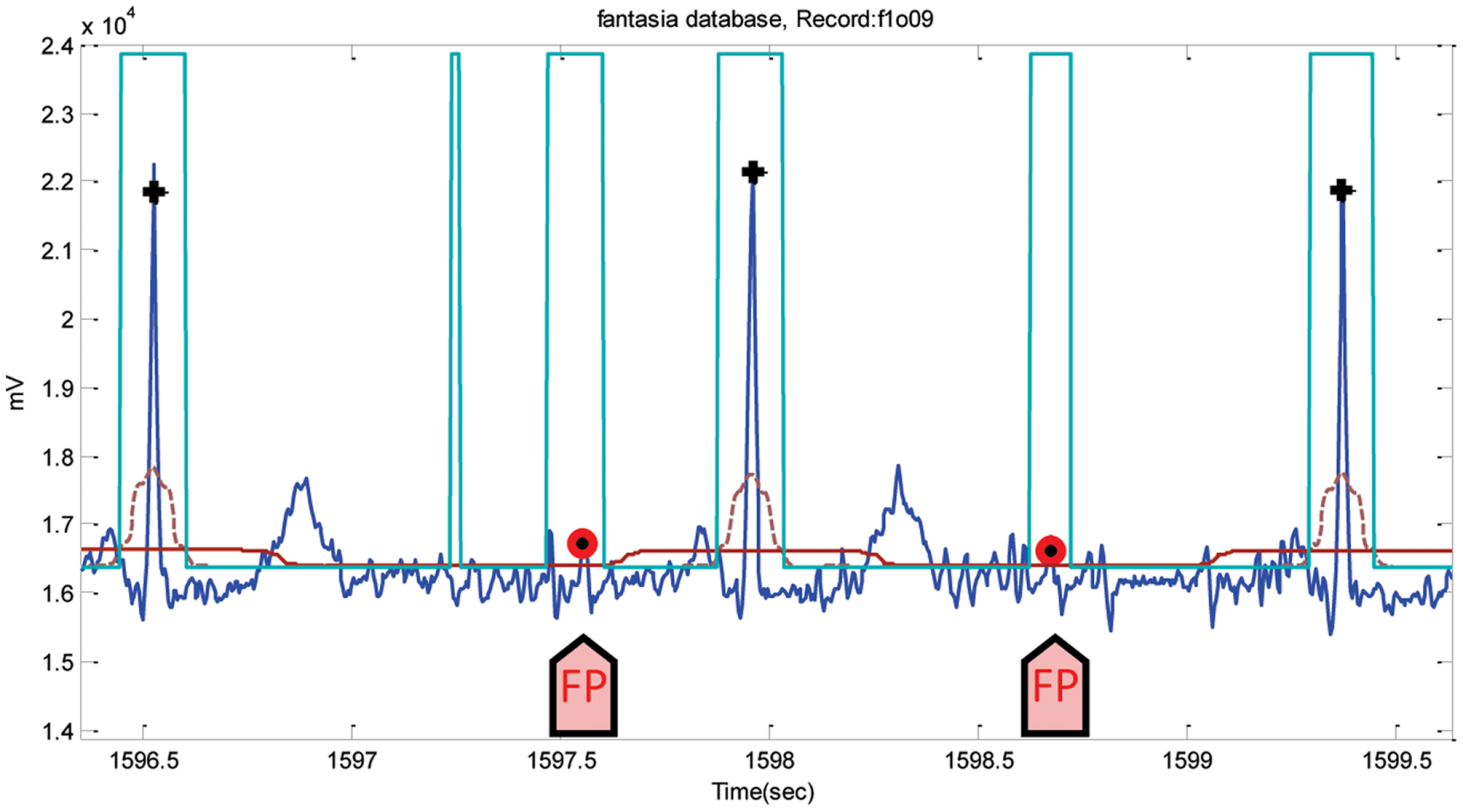
Wide U waves in Record f1o09-FANTASIADB. The dotted line is the first moving average 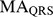, and the solid line is the second moving average 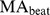. The arrows point to FPs. Here, the black plus sign represents successful detection produced by the proposed algorithm, where the red circle represents FP.

For the INCART database, the algorithm incurred a total of 5197 FPs and 1995 FNs. Because of the very noisy signals, Record 53 had 428 FPs and 104 FNs (cf. Figure 11). The annotations of this database may need revision as the position of the R peaks is very hard to determine, as shown in Figure 11. However, the algorithm runs over the database without any adjustments to the annotated R peaks. FPs and FNs were 315 and 50 when the algorithm was applied on the Fantasia database. The highest FP values were in record f1o09, where the ECG signals contain wide U waves, as shown in Figure 12. Likewise, Record 16272 (in the NSR database) had the most number of FPs, 49 instances out of 63 FPs, because of the existence of U waves. The algorithm incurred a total of 5197 FPs and 1995 FNs on INCART database. Because of the very noisy signals, Record 53 had 428 FPs, and 104 FNs (cf. Figure 11). The annotations of this database perhaps needs revision as the position of the R peaks is very hard to determine, as shown in Figure 11. However, the algorithm runs over the database without any adjustments to the annotated R peaks.

**Figure 13:**
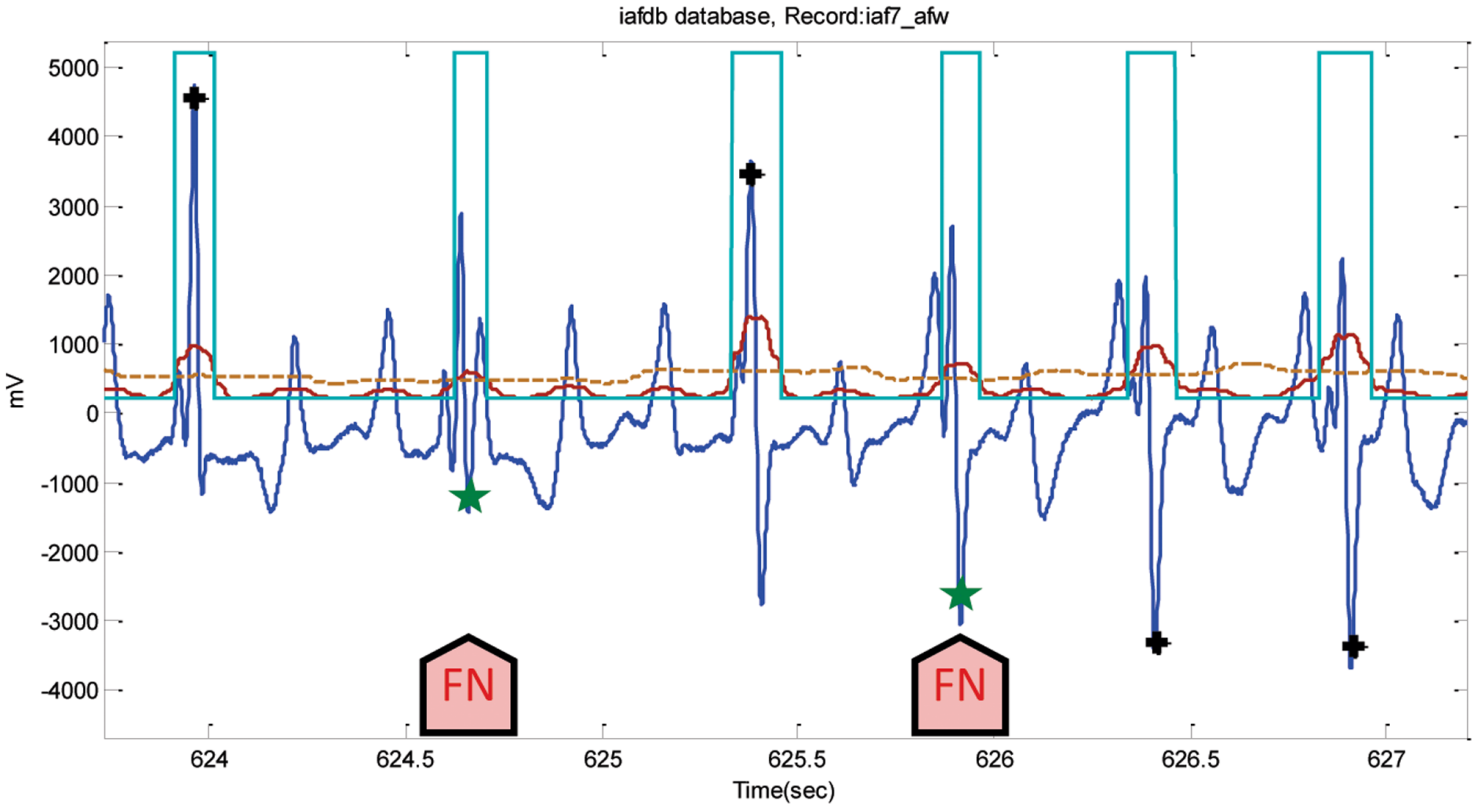
Isolated QRS-like artifacts in Record iaf7_afw-IAFDB. The dotted line is the first moving average 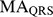, and the solid line is the second moving average 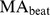. The arrows point to FNs. Here, the black plus sign represents successful detection produced by the proposed algorithm, where the green star represents FN.

Using the AFTDB database, the detector achieved a low number of FPs, and FNs, 17 and 34, correspondingly. Due to the fast rhythm of the atrial fibrillation, the number of FNs was higher than that of the FPs, which is similar to the detectors performance on the MIT-BIH Arrhythmia Database; Figure 10 may clarify the idea of the occurrence of FNs in a fast rhythm. It was expected that SVDBs performance would have more FNs than FPs, as it contains supraventricular arrhythmias. However, the highest number of FNs was registered from Record 848-SVDB due to the rapid heart rhythm. The number of FPs also increased because of the noisy reversed-polarity QRS beats, as in Record 886, which had the highest number of FPs, exactly 99 of a total of 356. Figure 13 shows how the isolated QRS-like artifacts caused FNs in Record iaf7_afw from the IAF database, scoring the highest number of FNs, 80 FNs out of a total of 83. On the other hand, the number of FPs was the highest, 250 out of a total 419 FPs, in Record iaf5_afw, which contains wide U waves similar to the example presented in Figure 12.

**Figure 14:**
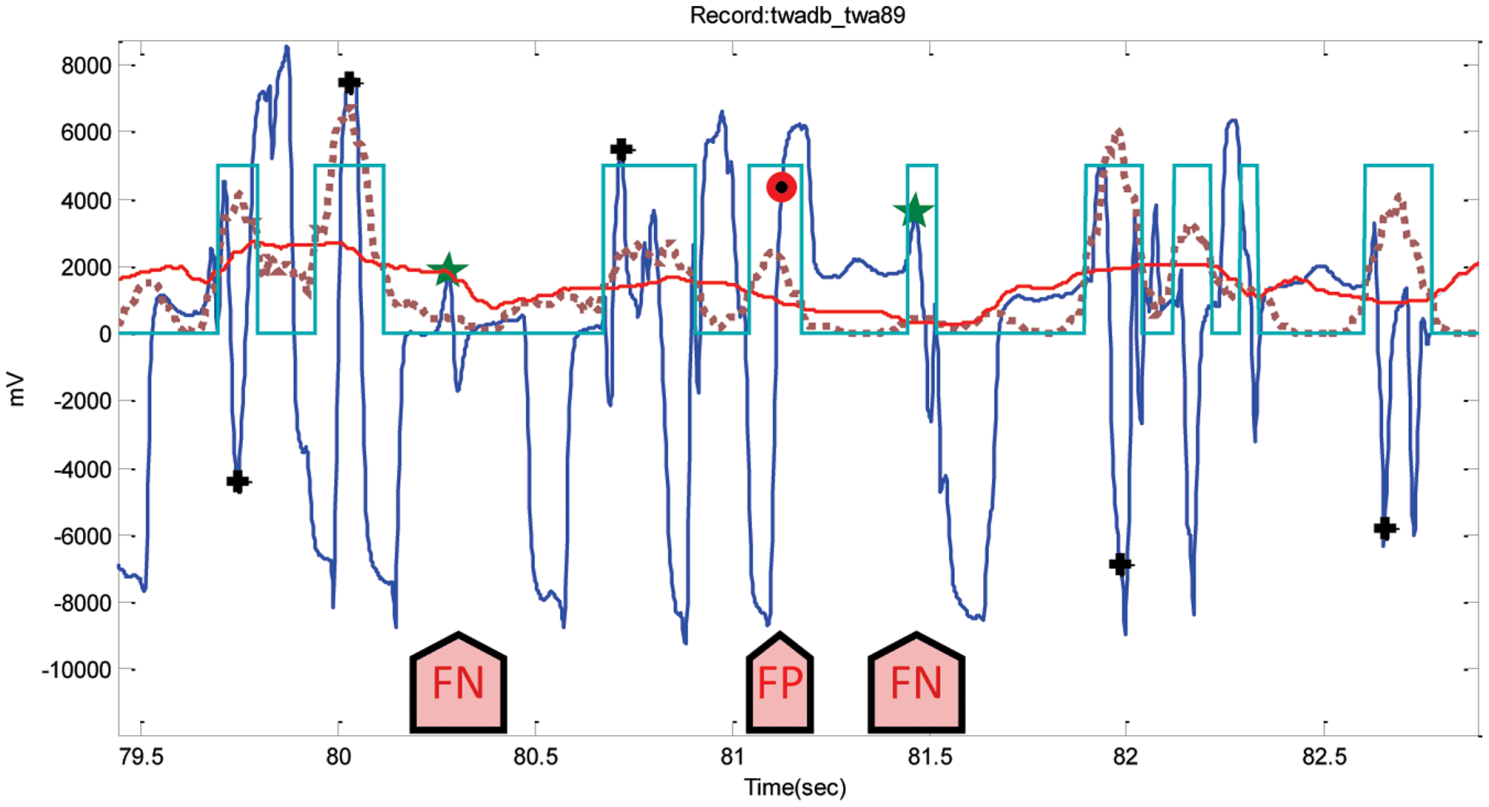
Low-amplitude QRS complexes lie between T wave alternans in Record twa89-TWADB. The dotted line is the first moving average 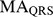, and the solid line is the second moving average 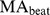. The arrows point to FNs and FP. Here, the black plus sign represents successful detection produced by the proposed algorithm, and the red circle represents FP, while the green star represents FN.

It can be seen in Figure 14, because of the T wave alternans and low-amplitude QRS complexes, that detecting R peaks is challenging. The performance of the detector on the TWA database incurred 156 FPs and 230 FNs. The first FN (at left) occurred because the moving average could not generate blocks of interest; however, the second FN (at right) happened since it has been demarcated (cf. Figure 14). The duration of the block (second FN at right) is below the optimized duration of QRS complex 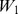, and is thus rejected causing FN, while the FP arises due to the existence of noisy T wave alternans.

**Figure 15:**
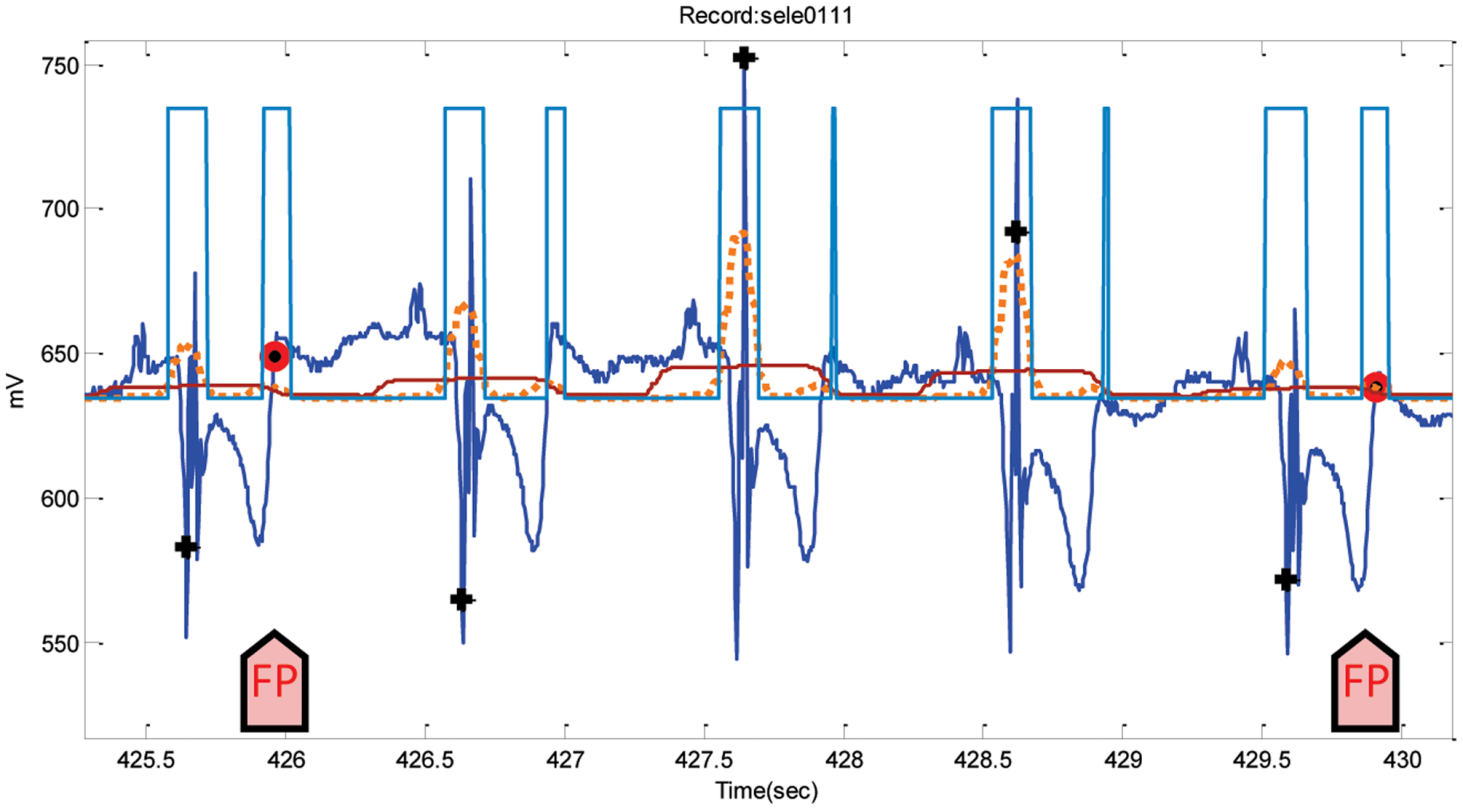
Steeply upward-sloping T waves in Record sele0111-QTDB. The dotted line is the first moving average 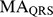, and the solid line is the second moving average 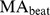. The arrows point to FPs. Here, the black plus sign represents successful detection produced by the proposed algorithm, and the red circle represents FP.

Analyzing the performance of NSTDB is quite confusing, perhaps because the annotations are not completely correct and certainly need modification. However, the detector ran over the dataset as it is and incurred 2,844 FPs and 1,199 FNs overall. Regarding the ST database, the FPs and FNs were 131 and 33 in total, respectively. The highest number of FPs occurred in Record 305-STDB due to large T waves, while the inverted polarity of QRS complexes caused the large number of FNs. On the other hand, the detectors obtained a total of 305 FPs and 3 FNs over the QT database. The FPs are mainly caused by the steeply upward-sloping T waves (cf. Figure 15).

### Processing Time

**Figure 16:**
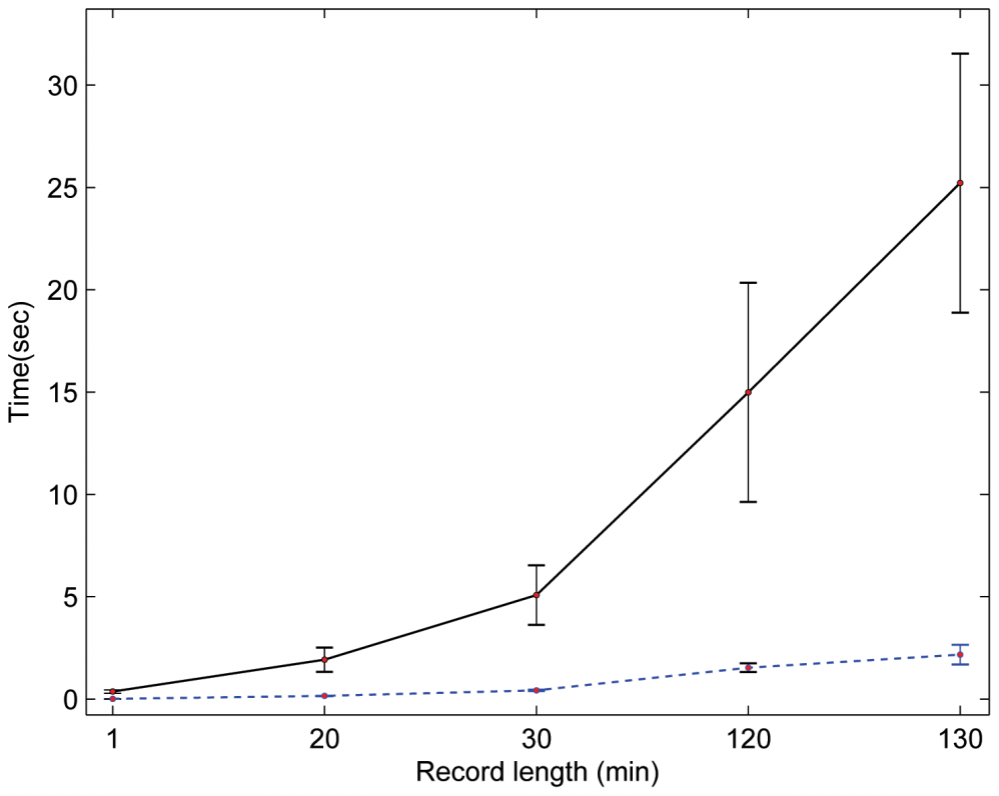
Processing time for ECG recordings. The average speed of the Pan and Tompkins algorithm is represented with a solid black line, while the dotted blue line represents the average speed of the proposed detector. The proposed detector processes the one-minute ECG record in 8.9 milliseconds and the 130-minute recording in 2.2 seconds. This result shows the superiority of the proposed detector over the Pan and Tompkins algorithm in terms of processing time.

In this study, the proposed detector was implemented in MATLAB 2010b (The MathWorks, Inc., Natick, MA, USA) on Intel™ i5 CPU 2.27 GHz. Perhaps it is misleading to suggest that mentioning the average speed of the proposed detector, over a certain time length of ECG signal, would provide a comparative result. This is because the processing time depends on the number of beats within each ECG recording, not on the record length. As the 11 databases contain different recording lengths, a categorization by recording length is needed to evaluate the speed of the Pan and Tompkins algorithm and the proposed detector fairly on the same computer. It can be seen in Figure 16 that the proposed algorithm was faster and steadier across all recoding-length categories compared to Pan and Tompkins algorithm. The speed measured in seconds, while the recording-length category was in minutes. The number of beats of the 30-minute recordings category was relatively consistent—with a mean ± SD, number of beats 2291±448—over all records of this category. The same holds for 1-minute and 15-minute recording categories. On the contrary, the 130-minute beat average was 10,171 with an SD of 2,600 beats; thus, the processing time depends on the number of beats rather than the recording length. For example, Record 16272-NSRDB contains 7,988 beats, and the proposed detector took 1.5 seconds to process it, while it took 3.5 seconds to process 14,875 beats in Record 19830-NSRDB. In general, without taking the number of beats into consideration, the speed of the proposed detector is fast. The suggested detector handles 15-minute recordings in about 0.15 seconds, while it takes about 2.2 seconds to handle 130-minute ECG recordings.

## Limitations of Study and Future Work

One of the next steps regarding the result of this study is to detect arrhythmic ECG beats, using the RR interval as the main feature. In addition, the detection of P and T waves based on the accurate detection of R peaks need to be examined.

Optimization was performed over the MIT-BIH Arrhythmia Database as a whole. For better comparability, the whole data set could have been separated into a training and a test set in which the training set is used for optimizing and the test set for evaluating the performance. Perhaps the leave-one-out cross validation suits this optimization step.

In this investigation, the processing time of the Pan and Tompkins algorithm and the proposed algorithm is carried out using MATLAB for batch processing of ECG files. MATLAB does only high precision floating point arithmetic. Perhaps, the implementation of the proposed algorithm in C-language is required to compare the performance over fixed point integer arithmetic (i.e., it doesn't need a high performance computer with a multi-precision floating point processor to run).

It is important to note that the largest ECG recording used in this study is 130 minutes. Larger recordings (e.g., 24 hours) with different noise levels are needed in order to generalize the findings of this study.

Technically, exploring the event-related moving average methodology for detecting events in ECG signals is promising in terms of computational complexity and efficiency. This can be further improved by investigating other band-pass filters, with different orders, and also by developing fast-moving average techniques for real-time analysis and mobile phone applications.

## Conclusion

A new approach for detecting QRS in ECG signals is presented. It contains two parts: the optimization, which was more complex, and the algorithm itself, which is tuned now and can be implemented and used with relative easiness. The performance of the optimized knowledge-based detector is promising. It has been tested on different databases that contain unusual noise, QRS, T, and U waves morphologies. The extensive use of the MIT-BIH Database as a testing database can hide overtuning of the detector parameters to fit this particular database. Consequently, the validation of the same detector on a second dataset without any later parameter tuning can help to obtain more reliable performance results. After the algorithm was applied on other databases, high detection rates were obtained on the QT database, NSR, TWA, IAF, ST, SV, AFT, FANTASIA, NST and ICART databases. Interestingly, the detector's speed over 130-minute recordings is about 2.2 seconds; thus, the proposed detector is an auspicious tool for processing large-recorded ECG signals. Furthermore, its simplicity makes it an ideal algorithm for mobile-phone applications and battery-driven ECG signal devices. Moreover, such a fast robust algorithm could have several interesting applications in an online analysis of cardiac data collected by the smallest long-term recording devices that have been studied in the form of necklaces and smart electrodes. The assessment of the QRS detector has been reliably conducted over the existing standard databases. Moreover, the number of annotated beats used in testing the new algorithm is considered sufficient as it is tested on a good representation of the possible morphologies found in ECG signals.

## References

[1] Alwan A (2011) Global status report on noncommunicable disaeses 2010. World Health Organization.

[2] Dilaveris PE, Gialafos EJ, Sideris SK, Theopistou AM, Andrikopoulos GK, et al (1998) Simple electrocardiographic markers for the prediction of paroxysmal idiopathic atrial fibrillation. American Heart Journal 135: 733–738. 9588401 10.1016/s0002-8703(98)70030-4

[3] Pan J, Tompkins W (1985) A real-time QRS detection algorithm. IEEE Trans Biomed Eng 32: 230–236. 3997178 10.1109/TBME.1985.325532

[4] Silva I, Moody GB, Celi L (2011) Improving the quality of ECGs collected using mobile phones: The Physionet/Computing in Cardiology Challenge 2011. In: Proc. IEEE Computing in Cardiology. pp. 273–276.

[5] Moody GB, Mark RG (2001) The impact of the MIT-BIH arrhythmia database. IEEE Engineering in Medicine and Biology Magazine 20: 45–50. 11446209 10.1109/51.932724

[6] Friesen G, Jannett T, Jadallah M, Yates S, Quint S, et al (1990) A comparison of the noise sensitivity of nine QRS detection algorithms. IEEE Trans on Biomed Eng 37: 85–98. 10.1109/10.43620 2303275

[7] Braunwald E, Zipes D, Libby P, Bonow R (2004) Braunwald's Heart Disease: A Textbook of Cardiovascular Medicine, volume Single Volume. Philadelphia: Saunders, 7th edition edition.

[8] Laguna P, Mark R, Goldberg A, Moody G (1997) A database for evaluation of algorithms for measurement of QT and other waveform intervals in the ECG. In: Proc. IEEE Computers in Cardiology 1997. pp. 673–676. doi:10.1109/CIC.1997.648140.

[9] Moody GB (2008) The Physionet/Computers in Cardiology challenge 2008: T-wave alternans. In: Proc. IEEE Computers in Cardiology. pp. 505–508. 10.1109/CIC.2008.4749089 PMC2749698 19779602

[10] Goldberger AL, Amaral LAN, Glass L, Hausdorff JM, Ivanov PC, et al (2000) PhysioBank, PhysioToolkit, and PhysioNet: Components of a new research resource for complex physiologic signals. Circulation 101: e215–e220. 10851218 10.1161/01.cir.101.23.e215

[11] Albrecht P (1983) S-T segment characterization for long-term automated ECG analysis. M.S. thesis, MIT Dept. of Electrical Engineering and Computer Science, Boston, MA, USA.

[12] Greenwald S (1990) Improved detection and classification of arrhythmias in noise-corrupted electro-cardiograms using contextual information. Ph.D. thesis, Harvard-MIT Division of Health Sciences and Technology, Boston, MA, USA.

[13] Moody G (2004) Spontaneous termination of atrial fibrillation: a challenge from Physionet and Computers in Cardiology 2004. In: Proc. IEEE Computers in Cardiology. pp. 101–104. doi: 10.1109/CIC.2004.1442881.

[14] Iyengar N, Peng CK, Morin R, Goldberger AL, Lipsitz LA (1996) Age-related alterations in the fractal scaling of cardiac interbeat interval dynamics. American Journal of Physiology - Regulatory, Integrative and Comparative Physiology 271: R1078–R1084. 10.1152/ajpregu.1996.271.4.R1078 8898003

[15] Moody GB, Muldrow W, Mark R (1984) A noise stress test for arrhythmia detectors. In: Proc. IEEE Computers in Cardiology. pp. 381–384.

[16] Clifford GD, Azuaje F, McSharry P (2006) Advanced Methods And Tools for ECG Data Analysis. Norwood, MA, USA: Artech House, Inc.

[17] Oppenheim A, Shafer R (1989) Discrete-time Signal Processing. NJ: Prentice Hall.

[18] Thakor NV, Webster JG, Tompkins WJ (1983) Optimal QRS detector. Medical and Biological Engineering 21: 343–50. 10.1007/BF02478504 6876910

[19] Chen H, Chen S (2003) A moving average based filtering system with its application to real-time QRS detection. In: Proc. IEEE Computers in Cardiology. pp. 585–588. doi: 10.1109/CIC.2003.1291223.

[20] Firbank M, Coulthard A, Harrison R, Williams E (1999) A comparison of two methods for measuring the signal to noise ratio on MR images. Physics in Medicine and Biology 44: 261–264. 10.1088/0031-9155/44/12/403 10616158

[21] Arzeno N, Deng Z, Poon C (2008) Analysis of first-derivative based QRS detection algorithms. IEEE Trans on Biomed Eng 55: 478–484. 10.1109/TBME.2007.912658 PMC2532677 18269982

[22] Elgendi M, Mahalingam S, Jonkman M, De Boer F (2008) A robust QRS complex detection algorithm using dynamic thresholds. In: Proc. IEEE Int. Symp. Computer Science and its Applications (CSA'08), Hobart, Tasmania, Australia, pp. 153–158.

[23] Elgendi M, Jonkman M, De Boer F (2009) Improved QRS detection algorithm using dynamic thresholds. International Journal of Hybrid Information Technology (IJHT) 2: 56–80.

[24] Adnane M, Jiang Z, Choi S (2009) Development of QRS detection algorithm designed for wearable cardiorespiratory system. Computer Methods and Programs in Biomedicine 93: 20–31. 18786742 10.1016/j.cmpb.2008.07.010

[25] Ghaffari A, Golbayani H, Ghasemi M (2008) A new mathematical based QRS detector using continuous wavelet transform. Computers & Electrical Engineering 34: 81–91.

[26] Zheng H, Wu J (2008) Real-time QRS detection method. In: Proc. IEEE 10th Int. Conf. Real-time QRS detection method, e-health Networking, Applications and Services (HealthCom 2008), Singapore, pp. 169–170. doi:10.1109/HEALTH.2008.4600130.

[27] Fard P, Moradi M, Tajvidi M (2007) A novel approach in R peak detection using hybrid complex wavelet (HCW). International Journal of Cardiology 124: 250–253. 17389145 10.1016/j.ijcard.2006.11.236

[28] Darrington J (2006) Towards real time QRS detection: a fast method using minimal pre-processing. Biomedical Signal Processing and Control 1: 169–176.

[29] Chen SW, Chen HC, Chan HL (2006) A real-time QRS detection method based on moving-averaging incorporating with wavelet denoising. Computer Methods and Programs in Biomedicine 82: 187–195. 16716445 10.1016/j.cmpb.2005.11.012

[30] Martinez JP, Almeida R, Olmos S, Rocha AP, Laguna P (2004) A wavelet-based ECG delineator: evaluation on standard databases. IEEE Transactions on Biomedical Engineering 51: 570–581. 15072211 10.1109/TBME.2003.821031

[31] Hamilton P (2002) Open source ECG analysis. In: Proc. IEEE Computers in Cardiology. pp. 101–104. doi:10.1109/CIC.2002.1166717.

[32] Lee J, Jeong K, Yoon J, Lee JH (1996) A simple real-time QRS detection algorithm. In: Proc. 18th Annual International Conference of the IEEE Engineering in Medicine and Biology Society, 1996. Bridging Disciplines for Biomedicine. volume 4, pp. 1396–1398. doi:10.1109/IEMBS.1996.647473.

[33] Afonso VX, Tompkins WJ, Nguyen TQ, Luo S (1996) Filter bank-based ECG beat detection. In: Proc. 18th Annual Int. Conf. IEEE Engineering in Medicine and Biology Society; Bridging Disciplines for Biomedicine, Amsterdam, Netherlands, Vol. 3, 1037–1038. doi: 10.1109/IEMBS.1996.652698.

[34] Li C, Zheng C, Tai C (1995) Detection of ECG characteristic points using wavelet transforms. IEEE Trans on Biomed Eng 42: 21–28. 10.1109/10.362922 7851927

[35] Hamilton PS, Tompkins WJ (1986) Quantitative Investigation of QRS Detection Rules Using the MIT/BIH Arrhythmia Database. IEEE Trans on Biomed Eng BME-33: 1157–1165. 10.1109/tbme.1986.325695 3817849

[36] Benitez DS, Gaydecki PA, Zaidi A, Fitzpatrick AP (2000) A new QRS detection algorithm based on the Hilbert transform. In: Proc. IEEE Computers in Cardiology. pp. 379–382.

[37] Xue Q, Hu Y, Tompkins W (1992) Neural-network-based adaptive matched filtering for QRS detection. IEEE Trans on Biomed Eng 39: 317–329. 10.1109/10.126604 1592397

[38] Moraes JCTB, Freitas MM, Vilani FN, Costa EV (2002) A QRS complex detection algorithm using electrocardiogram leads. In: Proc. IEEE Computers in Cardiology. pp. 205–208. doi: 10.1109/CIC.2002.1166743.

[39] Englese WAH, Zeelenberg C (1979) A single scan algorithm for QRS detection and feature extraction. In: Proc. IEEE Computers in Cardiology. pp. 37–42.

[40] Ligtenberg A, Kunt M (1983) A robust-digital QRS detection algorithm for arrhythmia monitoring. Computers and Biomed Res 16: 273–286. 10.1016/0010-4809(83)90027-7 6872535

[41] Alvarado C, Arregui J, Ramos J, Pallas-Areny R (2005) Automatic detection of ECG ventricular activity waves using continuous spline wavelet transform. In: Proc. 2nd International Conference on Electrical and Electronics Engineering. pp. 189–192. doi:10.1109/ICEEE.2005.1529605.

[42] Zhang F, Lian Y (2007) Novel QRS detection by CWT for ECG sensor. In: Proc. IEEE Biomedical Circuits and Systems Conference. pp. 211–214. doi:10.1109/BIOCAS.2007.4463346.

[43] Tompkins W (2012). DigiScope, https://courses.moodle.wisc.edu/prod/course/view.php?id=115.

[44] Sahambi JS, Tandon S, Bhatt RKP (1997) Using wavelet transforms for ECG characterization. An on-line digital signal processing system. IEEE Engineering in Medicine and Biology Magazine 16: 77–83. 9058586 10.1109/51.566158

[45] Mahmoodabadi SZ, Ahmadian A, Abolhasani MD (2005) ECG feature extraction using Daubechies wavelets. In: Proc. Fifth IASTED International Conference. pp. 343–348. 10.1109/IEMBS.2005.1615314 17281084

[46] Elgendi M, Jonkman M, De Boer F (2010) Frequency bands effects on QRS detection. In: Proc. International Joint Conference on Biomedical Engineering Systems and Technologies, BIOSIGNALS 2010, Valencia, Spain. pp. 428–431.

